# CAPZA1 modulates EMT by regulating actin cytoskeleton remodelling in hepatocellular carcinoma

**DOI:** 10.1186/s13046-016-0474-0

**Published:** 2017-01-16

**Authors:** Deng Huang, Li Cao, Shuguo Zheng

**Affiliations:** Institute of Hepatobiliary Surgery, Southwest Hospital, Third Military Medical University, No.29 Gaotanyan Road, Shapingba District, Chongqing, 400038 China

**Keywords:** CAPZA1, Actin, Primary hepatocellular carcinoma (HCC), Metastasis, Epithelial-mesenchymal transition (EMT)

## Abstract

**Background:**

Epithelial-mesenchymal transition (EMT) elicits dramatic changes, including cytoskeleton remodelling as well as changes in gene expression and cellular phenotypes. During this process, actin filament assembly plays an important role in maintaining the morphology and movement of tumour cells. Capping protein, a protein complex referred to as CapZ, is an actin-binding complex that can regulate actin cytoskeleton remodelling. CAPZA1 is the α1 subunit of this complex, and we hypothesized that CAPZA1 regulates EMT through the regulation of actin filaments assembly, thus reducing the metastatic ability of hepatocellular carcinoma (HCC) cells.

**Methods:**

Immunohistochemistry was used to detect CAPZA1 expression in 129 HCC tissues. Western blotting and qPCR were used to detect CAPZA1, EMT markers and EMT transcription factors in HCC cells. Transwell migration and invasion assays were performed to observe the migration and invasion of HCC cells. Cell Counting Kit-8 (CCK-8) was used to detect the proliferation of HCC cells. Immunoprecipitation was used to detect the interaction between CAPZA1 and actin filaments. Finally, a small animal magnetic resonance imager (MRI) was used to observe metastases in HCC cell xenografts in the liver.

**Results:**

CAPZA1 expression levels were negatively correlated with the biological characteristics of primary HCC and patient prognosis. CAPZA1 expression was negatively correlated with the migration and invasion of HCC cells. CAPZA1 down regulation promoted the migration and invasion of HCC cells. Conversely, CAPZA1 overexpression significantly inhibited the migration and invasion of HCC cells. Moreover, CAPZA1 expression levels were correlated with the expression of the EMT markers E-cadherin, N-cadherin and Vimentin. Furthermore, the expression of Snail1 and ZEB1 were negatively correlated with CAPZA1 expression levels. Similarly, CAPZA1 significantly inhibited intrahepatic metastases of HCC cells in an orthotopic transplantation tumour model.

**Conclusions:**

CAPZA1 inhibits EMT in HCC cells by regulating actin cytoskeleton remodelling, thereby reducing the metastatic ability of the cells. Together, our data suggest that CAPZA1 could be a useful biomarker for clinical determination of the prognosis of HCC patients.

## Background

Worldwide, hepatocellular carcinoma (HCC) is the sixth most commonly diagnosed cancer and the second leading cause of cancer-related mortality [1]. HCC is especially prominent in China, which accounts for 50% of HCC cases and deaths. Thus, HCC is a major disease affecting health in China [2]. In recent years, advances in comprehensive surgical treatments have achieved improved results, but relapse and metastasis still occur in 70% of patients within 5 years of surgery, seriously affecting treatment efficacy [3].

Epithelial-mesenchymal transition (EMT) is an important mechanism of tumour cell metastasis [4]. EMT refers to the biological process wherein epithelial cells gain a mesenchymal phenotype through specific changes in gene expression [5]. During this process, the epithelial cytoskeleton is restructured such that cell polarity and connections with the basement membrane are lost, resulting in increased metastatic capabilities [6]. Studies have shown that, as with many other cancer cells, HCC cells can obtain mesenchymal characteristics through EMT. This allows cells to detach from the primary lesion, invade blood vessels and colonize distant sites, forming metastatic lesions [7]. Thus, inhibiting metastasis by regulating EMT in HCC has become a key objective over recent years.

Actin is one of the most important components of the cytoskeleton, and changes in intracellular actin structures are closely related to EMT [8]. During EMT, the addition of G-actin to the barbed-end of existing actin filaments plays an important role in forming the cellular projections required for mesenchymal-type migration. Dynamic reorganization of the actin cytoskeleton is a precondition for tumour cell morphogenesis and metastasis [9, 10]. The actin-binding complex CapZ can bind to the barbed ends of actin filaments, and its expression is associated with the dynamic assembly of actin filaments and cell motility [11, 12]. CapZ consists of α and β subunits. α1 and α2 are found in chickens, mice and humans, and α3 is found only in murine testicular germ cells; the three β subunits (β1, β2, β3) are formed by alternative splicing. CAPZA1 encodes the α1 subunit of CAPZ [13] and has been reported to play a role in gastric cancer metastasis. However, neither the role of CAPZA1 in HCC nor the molecular mechanism of CAPZA1 regulation of tumour metastasis has been defined [14].

In this study, we assessed whether CAPZA1 regulates EMT by regulating actin cytoskeleton remodelling, thereby influencing the level of metastasis in HCC. Our hypothesis was made in accordance with existing research findings and tested using both in vitro and in vivo models.

## Methods

### Cases and follow-up

We collected 129 samples from HCC patients who received pathological liver resection at Southwest Hospital between January 2011 and December 2011. Clinicopathological data, including gender, age, tumour size, TNM stage, HCC differentiation, lymph node metastasis, vascular invasion, extrahepatic metastasis and other information, were collected from patient records and pathological examination. We conducted postoperative follow-up with patients until December 31, 2015; postoperative relapse and deaths were also recorded.

### Immunohistochemical staining

**Fig. 1:**
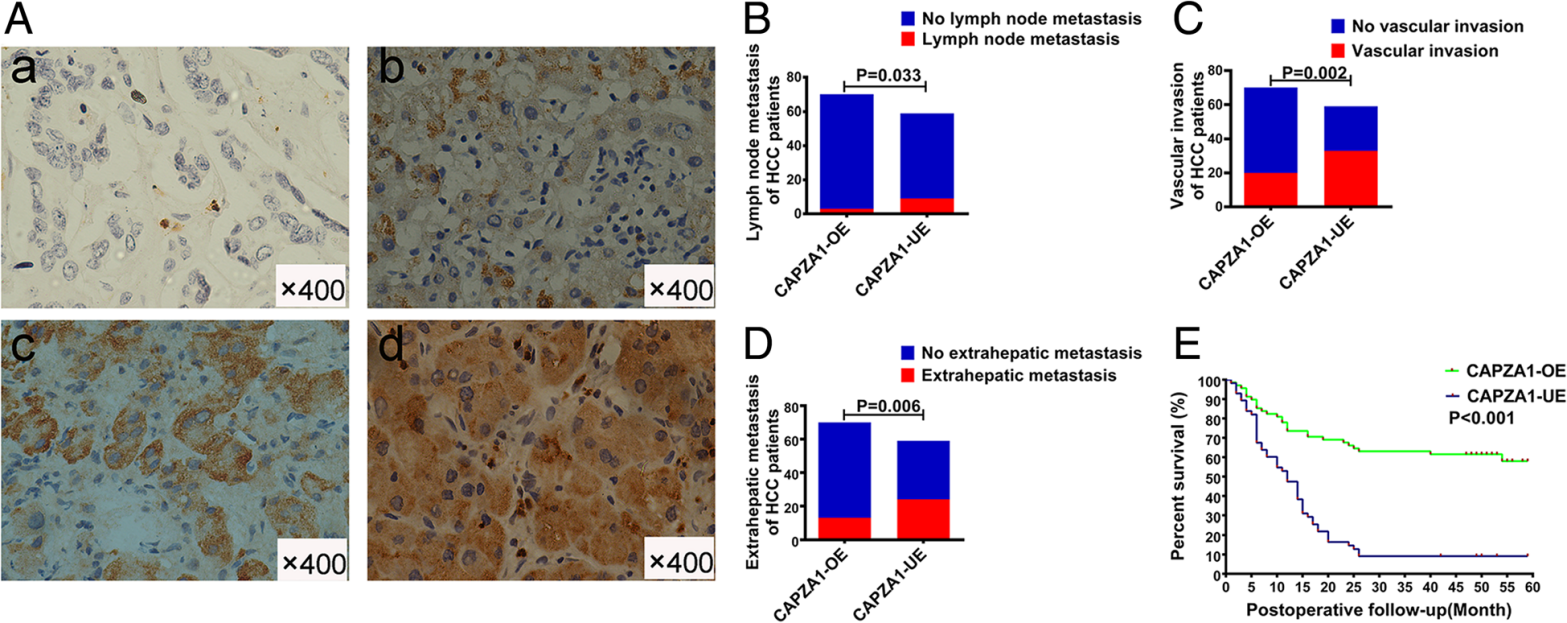
CAPZA1 expression is correlated with HCC malignancy. **a** CAPZA1 immunohistochemistry was performed on 129 HCC samples; CAPZA1expression was scored based on the percentage of tumour cells as described in the Methods. **b** Lymph node metastases in HCC patients of the CAPZA1 overexpression and underexpression groups; red indicates the occurrence of lymph node metastases, and blue indicates no lymph node metastases. **c** Vascular invasion in HCC patients of the CAPZA1 overexpression and underexpression groups; red indicates the occurrence of vascular invasion, and blue indicates no vascular invasion. **d** Extrahepatic metastases in HCC patients of the CAPZA1 overexpression and underexpression groups; red indicates the occurrence of extrahepatic metastases, and blue indicates no extrahepatic metastases. **e** Postoperative survival times of HCC patients of the CAPZA1 overexpression and underexpression groups were analysed via Kaplan–Meier curves. The mean postoperative survival time of the CAPZA1 overexpression group (40.4 ± 2.9 months) was significantly higher than the underexpression group (14.6 ± 2.0 months; *P* < 0.01)

HCC tissue specimens were paraffin-embedded and sectioned. After sections were deparaffinized in water, they were placed in sodium citrate solution in a microwave oven at moderate heat for 10 min for antigen retrieval. After naturally cooling, the sections were incubated in 3% H_2_O_2_ for 10 min and then blocked in 10% goat serum at room temperature for 1 h. Anti-CAPZA1 polyclonal rabbit antibody (1:50 dilution; Proteintech, Rosemont, IL, USA) was added and incubated with the tissues overnight at 4 °C. CAPZA1 immunoreactivity was detected using an anti-mouse/rabbit universal immunohistochemical detection kit (Proteintech). Finally, the sections were dehydrated, after 2 min of haematoxylin staining, and mounted with neutral resin. CAPZA1 staining results were scored by two independent pathologists and grouped according to the percentage of positively stained HCC cells: 0 (0%), 1+ (1–24%), 2+ (25–49%), 3+ (50–74%), 4+ (75–100%) (Fig. 1a).

### Cell culture

HepG2 and MHCC97H cell lines (from our laboratory’s cell bank) were cultured in DMEM (Thermo Fisher Scientific, Waltham, MA, USA) containing 10% foetal bovine serum (Biological Industries, Cromwell, CT, USA) and were kept in a humidified incubator containing 5% CO_2_ at 37 °C.

### RNA interference

A small interfering RNA (siRNA) against CAPZA1 (si-CAPZA1; 5′-GGA ACA AGA UAC UCA GCU A-3′) was purchased from Biomics Biotech (Nantong, China). HepG2 cells were inoculated in 6-well plates 24 h before transfection to achieve 30–50% confluence. Lipofectamine 2000 transfection reagent (Invitrogen, Carlsbad, CA, USA) was used to transfect cells with the constructs. Cells were harvested after 24 h, and knockdown efficiency was verified by western blot.

### Lentiviral infection

Lentiviruses carrying interference (LV10-CAPZA1) and overexpression (LV8-CAPZA1) constructs for human CAPZA1 were built by (GenePharma, Shanghai, China). Cells were inoculated in 6-well plates 24 h prior to infection to achieve 50–70% confluence. For infection, 1 ml of fresh medium containing 10 μl of lentivirus (1 × 10^9^TU/ml) was added to each well, and after 72 h, the infection rate was observed using a fluorescence microscope, and CAPZA1expression was detected by western blot.

### Migration and invasion assay

Before invasion assays, 30 μl of Basement Membrane Matrix (diluted 1:6 in PBS; Corning Life Science, Lowell, CA, USA) was applied to the bottom of a Transwell chamber (Millipore, Billerica, MA, USA) and placed in a 37 °C incubator for 5 h. The following steps were the same for the migration and invasion assays: cells (6 × 10^4^) in serum-free media were added into the upper well of the Transwell chamber, and 800 μl of DMEM containing 10% FBS was added to the lower chamber, and then, the chamber was placed in a 37 °C incubator. After18h for migration assays or 36 h for invasion assays, cells were fixed with paraformaldehyde for 30 min, and stained for 3 min with crystal violet. Excess stain was washed away with PBS, and the bottom of the upper chamber was gently wiped with a cotton swab. After drying, the chamber was imaged, and the number of invading and migrating cells was calculated.

### Cell proliferation assay

Cells (1 × 10^3^) were inoculated into 96-well plates in triplicate, and the number of cells was detected at 0 h, 24 h, 48 h and 72 h. A Cell Counting Kit-8 (CCK-8; Engreen, Auckland, New Zealand) was used to detect cell numbers. Briefly, 10 μl of CCK-8 was added per well and incubated for 1 h, and then a microplate reader was used to detect absorbance at 450 nm.

### Western blotting and quantitative PCR (qPCR)

Total cellular protein was isolated in RIPA lysis buffer (CWBIO, Beijing, China), and protein concentration was detected using a BCA Protein Assay Kit (CWBIO). Western blotting was performed as previously described [15]. Antibodies against E-cadherin, N-cadherin and Vimentin were purchased from Proteintech. For qPCR assays, an ultrapure RNA Kit (CWBIO) was used to extract total RNA. The primers used to amplifyCAPZA1, E-cadherin, N-cadherin, vimentin and GAPDH areshown in Table 1. SYBR Premix Ex Taq II (Tli RNaseH Plus) (TaKaRa, Shiga, China) was used for reverse transcription and amplification, according to the manufacturer’s protocols.

**Table 1:** Primers and product size

Gene	Primers(5′-3′)	Product Size(bp)
h-CAPZA1-F	AATGAAGCCCAAACTGCCAA	158
h-CAPZA1-R	TTCCAGTCGATTTTGGTGCG	
h-E-cadherin-F	AACAGGATGGCTGAAGGTGA	192
h-E-cadherin-R	CCTTCCATGACAGACCCCTT	
h-N-cadherin-F	ATATTTCCATCCTGCGCGTG	195
h-N-cadherin-R	GTTTGGCCTGGCGTTCTTTA	
h-vimentin-F	GGACCAGCTAACCAACGACA	178
h-vimentin-R	AAGGTCAAGACGTGCCAGAG	
h-GAPDH-F	AGGGGCCATCCACAGTCTTC	258
h-GAPDH-R	AGAAGGCTGGGGCTCATTTG	

### Immunoprecipitation

A SureBeads reagent (BIO-RAD, California, USA) was thoroughly resuspended in its solution and 100 μl of SureBeads was transfered to 1.5 ml tubes. The beads were magnetized with magnetic separation rack and the supernatant was discarded. After washed with 1,000 μl PBST three times, the beads were resuspended with 200 μl final volume solution containing 3 μg of CAPZA1 antibody or actin antibody (Proteintech), and the total solution was rotated at least 30 min at room temperature. And then, the beads were magnetized and the supernatant was discarded. After the beads were washed with PBST three times, the beads were resuspended with 500 μl of the antigen-containing lysate, and the tubes were rotated for the night at 4 °C. The next morning, the beads were magnetized and the supernatant was discarded. After washed with PBST three times, the beads were resuspended with 30 μl SDS-PAGE loading buffer, and then, all tubes were boiled for 10 min. The residual buffer was aspirated from the tubes to the new one after the beads were magnetized. The rest of operation referenced the western blotting protocols.

### Xenograft models

HCC cells (sh-CAPZA1-expressing and sh-control-expressing HepG2 cells, CAPZA1-overexpressing and control MHCC97H cells) were harvested with trypsin and resuspended in DMEM. Nude mice were anesthetized with 1% sodium pentobarbital (100 ml/kg; Sigma-Aldrich, St. Louis, MO, USA), and 1 × 10^5^ HCC cells were injected under the liver capsule. After 6 weeks, a 7.0 T small animal MRI (Bruker Biospec, Ettlingen, Germany) was used to scan the chest and abdomen of the mice to observe metastases of the orthotopic HCC cells. The time of death of the mice was observed and recorded. After death, the liver and lungs were removed, and the surfaces were observed; then, the liver was sectioned, paraffinized and stained with haematoxylin and eosin, and tumour lesions were observed.

### Statistical analysis

Statistical analyses were conducted using SPSS 19.0 (SPSS Inc., Chicago, IL, USA) and Prism 6 (GraphPad, La Jolla, CA, USA), and all statistical tests were two-way. *P* < 0.05 was considered statistically significant.

## Results

### Patient demographics

Tissue samples from 129 HCC patients were collected for this study; the patient cohort had an average age of 47.8 ± 10.7 years and contained 115 men (89.1%). Average tumour size was 7.4 ± 3.2 cm (range: 1.0–18.0 cm). By TNM staging the patients were divided into stage I: 18.8% (*n* = 26), stage II: 9.3% (*n* = 13), stage III: 47.7% (*n* = 61) and stage IV: 24.2% (*n* = 29). By HCC differentiation the patients were divided into well differentiated: 10.4% (*n* = 10), moderately differentiated: 73.3% (*n* = 94), and poorly differentiated: 16.3% (*n* = 25). 9.3% (*n* = 12) of patients had lymph node metastasis, 44.1% (*n* = 53) of patients had vascular invasion, 28.7% (*n* = 37) of patients had extrahepatic metastasis before or after surgery, 70.5% (*n* = 91) of patients had recurrence within 5 years of surgery, 62.8% (*n* = 81) of patients died from cancer-related deaths within 5 years of surgery, and 3.1% (*n* = 4) of patients died from non-cancer-related deaths (Table 2).

**Table 2:** Clinicopathological and follow-up data from patients

Pathologic variables	No.of patients
TNM stage	129
Stage I	26(20.2%)
Stage II	13(10.1%)
Stage III	61(47.3%)
Stage IV	29(22.5%)
HCC differentiation	129
WD	10(7.8%)
MD	94(72.9%)
PD	25(19.4%)
Lymph node metastasis	129
Yes	12(9.3%)
No	117(90.7%)
Vascular invasion	129
Yes	53(41.1%)
No	76(58.9%)
Extrahepatic metastasis	129
Yes	37(28.7%)
No	92(71.3%)
Postoperative recurrence	129
Yes	91(70.5%)
No	38(29.5%)
Cancer related death	125
Death	81(64.8%)
Survival	44(35.2%)
CAPZA1 expression tatus	129
0	13(10.1%)
1+	46(35.7%)
2+	29(22.5%)
3+	26(20.2%)
4+	15(11.6%)

*HCC* hepatocellular carcinoma, *WD* well differentiated, *MD* moderately differentiated, *PD* poorly differentiated, *CAPZA1* capping protein α1 subunit

### CAPZA1 expression is negatively correlated with the biological characteristics of primary HCC and patient prognosis

CAPZA1 immunohistochemistry was conducted on the 129 HCC tissue samples and scored according to staining intensity. Based on these metrics, samples were divided into the following scoring groups: 0 accounted for 10.1% (*n* = 13) of patients, 1+ accounted for 35.7% (*n* = 46), 2+ accounted for 22.5% (*n* = 29), 3+ accounted for 20.2% (*n* = 26), and 4+ accounted for 11.6% (*n* = 15). Patients who scored 0 and 1+ were defined as the CAPZA1 underexpression group, and patients who scored 2+ and above were defined as the CAPZA1 overexpression group (Fig. 1a). We then analysed differences in TNM staging and HCC differentiation and compared the incidence of lymph node and vascular invasion, as well as patient prognosis, in the two groups. By TNM staging, the percentage of patients with stage I or II disease was significantly lower in the CAPZA1 underexpression group (3.4%) than in the CAPZA1 overexpression group (52.9%); conversely, the percentage of patients with stage III or IV disease was significantly higher in the CAPZA1 underexpression group (96.6%) than in the CAPZA1 overexpression group (47.2%; *P* < 0.001). By HCC differentiation class, the differentiation in the CAPZA1 underexpression group was poorer than in the CAPZA1 overexpression group (*P* < 0.001). The CAPZA1 underexpression group also had a higher rate of lymphoid tissue invasion (15.3% vs 4.3%; *P* = 0.033) (Fig. 1b), vascular invasion (55.9% vs 28.6%; *P* = 0.002) (Fig. 1c) and extrahepatic metastasis (40.7% vs 18.6%; *P* = 0.006) (Fig. 1d) compared with the overexpression group. Additionally, postoperative follow-up showed that the recurrence rate within 5 years of surgery in the underexpression group was significantly higher than in the CAPZA1 overexpression group (*P* < 0.001) (Table 3). Finally, Kaplan–Meier analysis showed that the mean postoperative survival time in the CAPZA1 underexpression group (14.6 ± 2.0 months) was significantly lower than in the CAPZA1 overexpression group (40.4 ± 2.9 months) (Fig. 1e).

**Table 3:** Comparison of the clinicopathological features and Patient prognosis in the CAPZA1 underexpression and overexpression

	Levels of CAPZA1 expression		P-value
	Underexpression 0,1+	Overexpression 2+,3+,4+	
Mean tumor size(cm)	8.0 ± 2.4	6.8 ± 3.6	0.032
TNM stage			<0.001
StageI	2(3.4%)	24(34.3%)	
StageII	0(0%)	13(18.6%)	
StageIII-IV	57(96.6%)	33(47.2%)	
HCC differentiation			<0.001
WD	0(0%)	10(14.3%)	
MD	36(61.0%)	58(82.9%)	
PD	23(39.0%)	2(2.9%)	
Lymph node metastasis	9/59(15.3%)	3/70(4.3%)	0.033
Vascular invasion	33/59(55.9%)	20/70(28.6%)	0.002
Extrahepatic metastasis	24/59(40.7%)	13/70(18.6%)	0.006
Postoperative recurrence	56/59(94.9%)	35/70(50%)	<0.001
Cancer related death	54/59(91.5%)	27/70(38.6%)	<0.001

*HCC* hepatocellular carcinoma, *WD* well differentiated, *MD* moderately differentiated, *PD* poorly differentiated, *CAPZA1* capping protein α1 subunit

### CAPZA1 inhibits HCC cell migration and invasion but does not inhibit proliferation

**Fig. 2:**
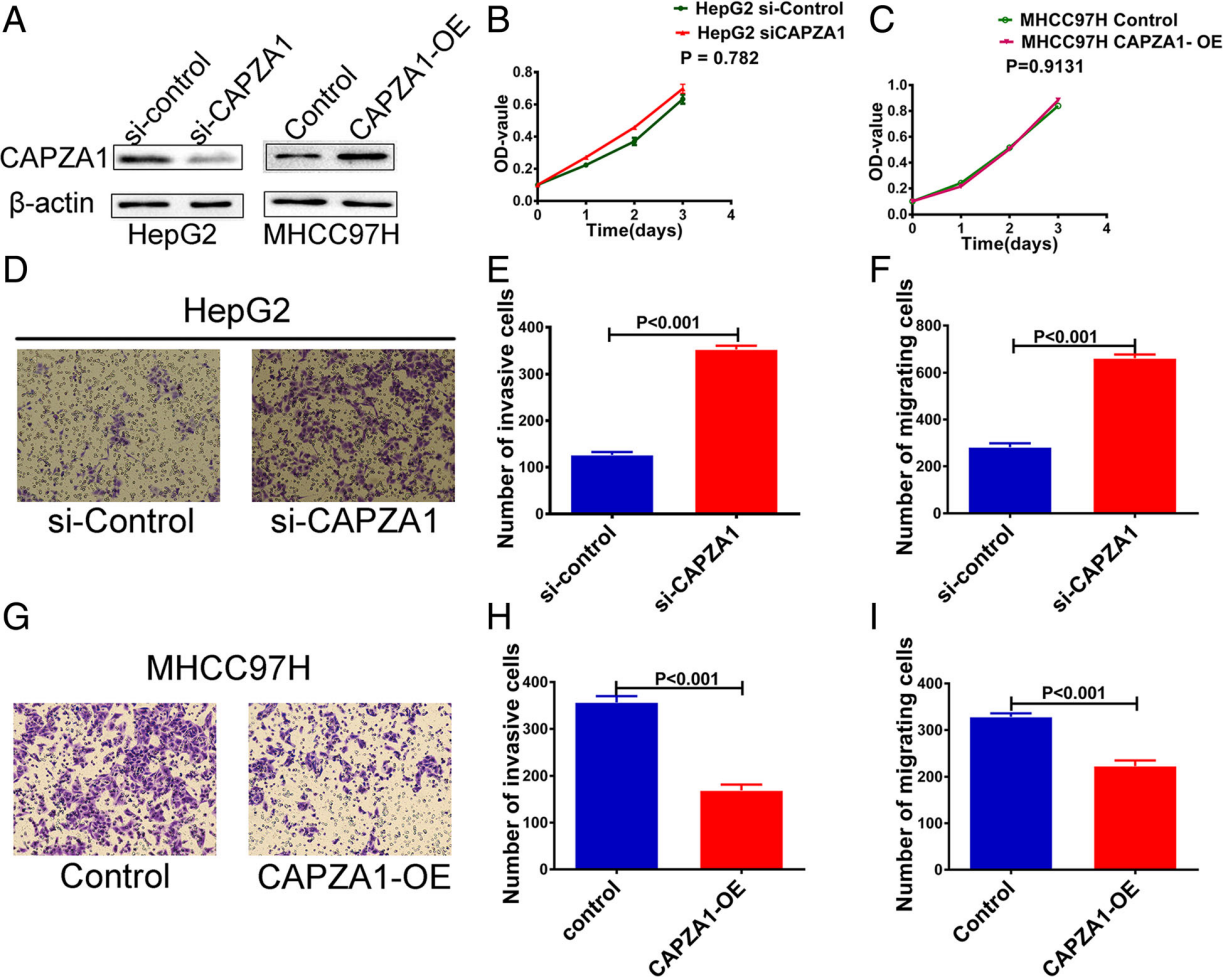
CAPZA1 expression controls metastasis of HCC cells. **a** Western blots to detect CAPZA1 expression in HepG2 cells 24 h after siRNA transfection and in MHCC97H cells 72 h after lentiviral-mediated overexpression. **b**, **c** A CCK-8 assay was used to detect the proliferation of HepG2 cells after siRNA-mediated knockdown of CAPZA1 (**b**) and MHCC97H cells following CAPZA1 overexpression (**c**). **d**–**f** Transwell migration and invasion assays were used to detect the migration and invasion potential of HepG2cells. After 36 h for invasion assays, HepG2 invasion was enhanced by CAPZA1 siRNA (**d**, **e**), and after 18 h for migration assays, HepG2 migration was enhanced by CAPZA1 siRNA (**f**). **g–i** Transwell migration and invasion assays were used to detect the migration and invasion potential of MHCCP7H cells. After 36 h for invasion assays, MHCC97H invasion was reduced by CAPZA1-overexpression (**g**, **j**), and after 18 h for migration assays, MHCC97H migration was reduced by CAPZA1-overexpression (**i**)

To test the effects of CAPZA1 expression on the proliferation, migration and invasion of HCC cells, we knocked down CAPZA1 with siRNA in HepG2 cells, which have weak metastatic potential (Fig. 2a). A CCK-8 assay was used to detect the proliferation of HepG2 cells transfected with control siRNA or CAPZ1 siRNA. The data showed that CAPZA1 down regulation had no effect on HepG2 proliferation (Fig. 2b). However, the results from Transwell migration and invasion assays showed that HepG2 migration and invasion were enhanced by CAPZA1 siRNA (Fig. 2d–e).

To further verify these effects, we constructed a CAPZA1 overexpression lentivirus and infected MHCC97H cells, which have strong invasion capabilities (Fig. 2a). Consistent with the results from the siRNA experiments, CAPZA1 overexpression had no effect on the proliferation of MHCC97H cells (Fig. 2c), but significantly reduced migration and invasion compared with control MHCC97H cells (Fig. 2g–i).

### CAPZA1 inhibits metastasis of HCC cells in nude mice

**Fig. 3:**
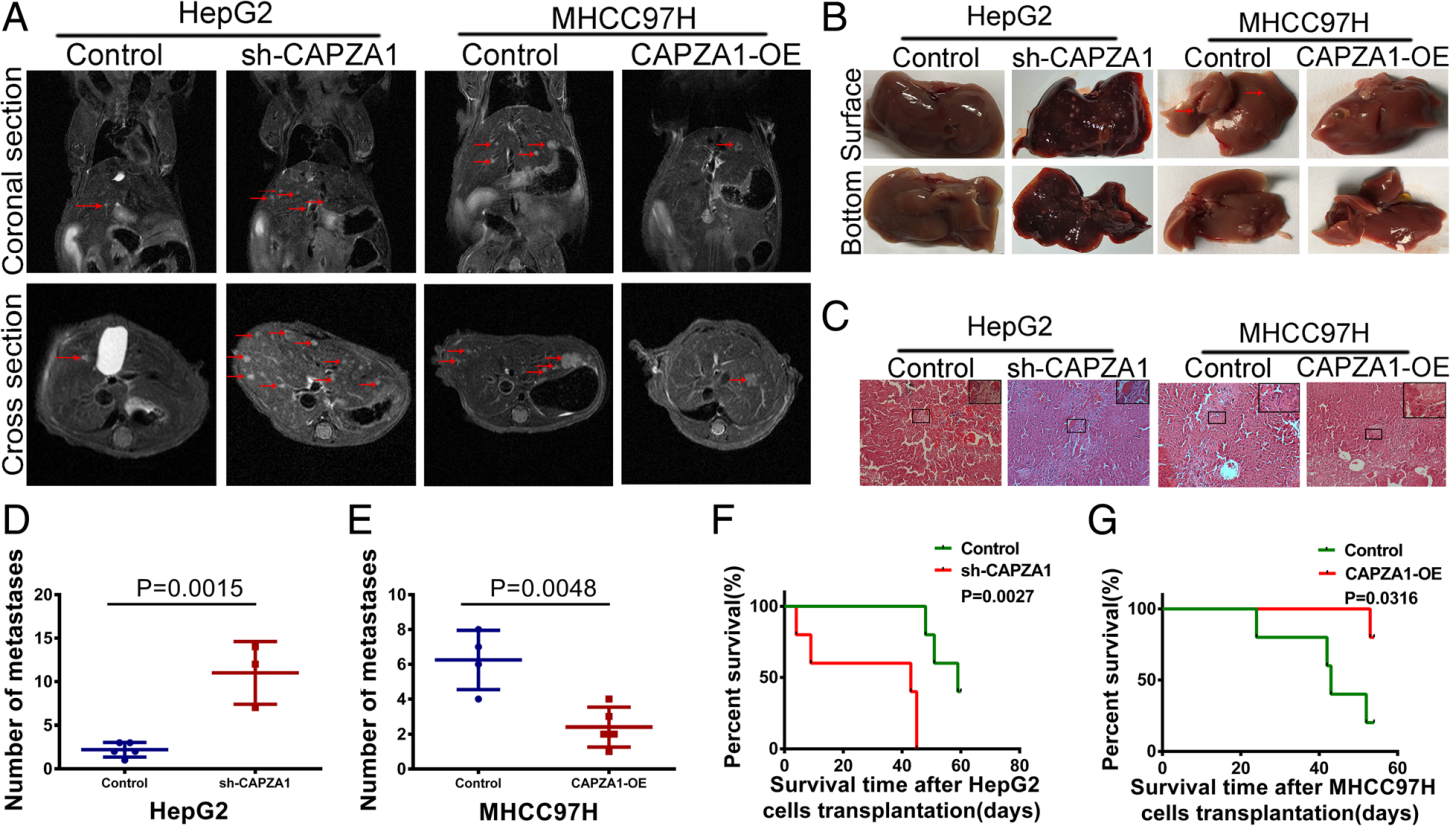
CAPZ1 expression controls *in vivo* tumour growth. **a** Chest and abdomen MRI scans of nude mice after 6 weeks of orthotopic hepatoma xenograft growth. The livers of nude mice in the sh-CAPZA1-expressing HepG2 group and control MHCC97H group showed obvious metastatic lesions, but few metastatic lesions were found in the sh-control-expressing HepG2 group and CAPZA1-overexpressing MHCC97H group; no lung metastases occurred in the four groups. **b** There were many metastatic lesions on the liver surface of the sh-CAPZA1-expressing HepG2 group and control MHCC97H group, but the liver surface of the sh-control-expressing HepG2 group and CAPZA1-overexpressing MHCC97 group were smooth, and showed no metastatic lesions. **c** Haematoxylin and eosin staining was performed on xenograft sections. **d**, **e** The number of lesions was counted on the MRI cross sections. The number of metastatic lesions in the sh-CAPZA1-expressing HepG2 group was significantly higher than in the sh-control-expressing HepG2 group (**d**). The number in control MHCC97H group metastatic lesions was significantly higher than in the CAPZA1-overexpressing MHCC97 group (**e**). **f**, **g** Survival analysis of the mice after HCC cell transplantation. The survival time of mice in the sh-control-expressing HepG2 group was longer than that of mice in the sh-CAPZA1-expressing group (**f**). In the CAPZA1-overexpressing MHCC97H group survival time was longer than in the control MHCC97H group (**g**)

In vitro experiments verified that CAPZA1 inhibits the migration and invasion of HCC cells. To verify these results in vivo, we constructed stable sh-CAPZA1- expressing HepG2 cells, CAPZA1-overexpressing MHCC97H cells and corresponding control cells in vitro and grew them in the liver capsule of nude mice. After six weeks, a small animal MRI was used to scan the chest and abdomen of the mice, and the results showed that extensive intrahepatic metastases occurred in 3 of 5 (60%) mice in the sh-CAPZA1-expressing HepG2 cells group, while a few intrahepatic metastases were found in each of the 5 mice in the sh-control-expressing HepG2 cells group; no pulmonary metastases occurred in either HepG2 groups (Fig. 3a). Similarly, in the MHCC97H cell-treated nude mice, a few intrahepatic metastases were found in the CAPZA1-overexpressing group of 5 nude mice, and extensive intrahepatic metastases were observed in 4 of 5 mice (80%) in the control MHCC97H group (Fig. 3a). The liver surface of mice in the sh-CAPZA1-expressing HepG2 group and MHCC97H control group were covered with micro-hepatoma lesions (Fig. 3b). The haematoxylin and eosin-stained nude mice hepatic sections revealed that the number of liver lesions in the sh-CAPZA1-expressing HepG2 group and MHCC97H control group were significantly increased compared with the sh-control-expressing HepG2 group and CAPZA1-overexpressing MHCC97H group (Fig. 3c-e). Finally, the survival time of mice in the sh-control-expressing HepG2 group was significantly longer than that of mice in the sh-CAPZA1-expressing HepG2 group (Fig. 3f). Meanwhile, the survival time of CAPZA1-overexpressing MHCC97H group mice was significantly longer than that of mice in the MHCC97H control group (Fig. 3g).

### CAPZA1 inhibits EMT in HCC cells by regulating actin cytoskeleton remodelling

**Fig. 4:**
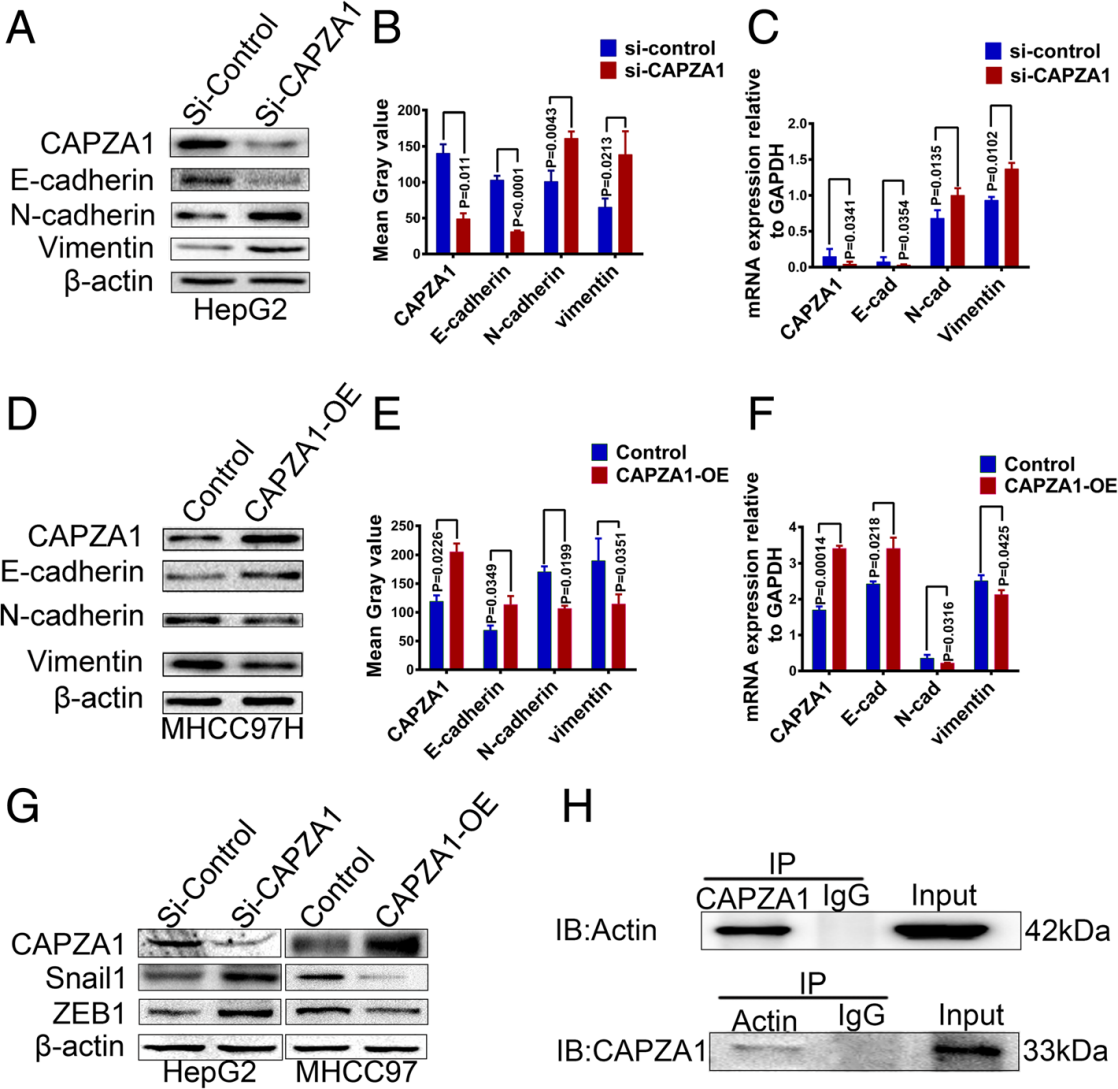
CAPZA1 expression controls HCC EMT. **a**, **b** Following CAPZA1 siRNA treatment, western blotting was used to detect the expression of EMT-related markers in HepG2 cells, and ImageJ software was used to measure immune reactivity. The epithelial marker E-cadherin was down-regulated, and the mesenchymal markers N-cadherin and Vimentin were up-regulated following CAPZA1 down regulated. **c** Following CAPZA1 siRNA treatment, qPCR was used to detect the mRNA expression of EMT-related markers in HepG2 cells. The results were consistent with the western blots in **a** and **b. d**, **e** Following CAPZA1 overexpression, western blotting was used to detect the expression of EMT-related markers in MHCC97H cells, and ImageJ software was used to measure immune reactivity. The epithelial marker E-cadherin was up-regulated, and the mesenchymal markers N-cadherin and Vimentin were down-regulated following CAPZA1 overexpression. **f** Following CAPZA1 overexpression, qPCR was used to detect the mRNA expression of EMT-related markers in MHCC97H cells. The results were consistent with the western blots in D and E. **g** EMT transcription factors were detected. Following treatment of HepG2 cells with CAPZA1 siRNA, Snail1 and ZEB1 expression was up-regulated. Following CAPZA1 overexpression in MHCC97H cells, expression of Snail1 and ZEB1 was down-regulated. **h** Immunoprecipitation was performed to validate interaction between CAPZA1 and F-actin. The result showed that CAPZA1 and actin can pull each other down

Actin filaments are an important component of the cytoskeleton that play a critical role in maintaining cell structure and regulating cell movement [16]. CAPZA1 is an actin-binding protein that is intimately involved in actin filament assembly [17]. To investigate whether CAPZA1 regulates EMT in HCC cells, we conducted siRNA and overexpression experiments and measured changes in the expression of the EMT markers E-cadherin, N-cadherin and Vimentin. When CAPZA1 expression was down-regulated, E-cadherin expression was decreased, while the mesenchymal markers N-cadherin and Vimentin were up-regulated (Fig. 4a–c); in contrast, when CAPZA1 was overexpressed, E-cadherin expression was up-regulated, while N-cadherin and Vimentin were down-regulated (Fig. 4d–f). Thus, CAPZA1 significantly inhibited EMT in HCC cells. To explore which transcription factor is involved in the EMT process, we detected EMT transcription factors of Snail family, Twist family and ZEB family. As the results, we found that the expression of Snail1 and ZEB1 were up-regulated when CAPZA1 expression was down-regulated. Conversely, the expression of Snail1 and ZEB1 were down-regulated when CAPZA1 was overexpressed (Fig. 4g). We also detected the interaction between CAPZA1 and F-actin, and the immunoprecipitation assay showed that CAPZA1 and actin can pull each other down (Fig. 4h). Thus, CAPZA1 inhibits EMT by regulating actin cytoskeleton remodeling via Snail1/ZEB1.

## Discussion

The high metastatic capabilities of HCC cells are the reason for the poor prognosis and high mortality rates of primary HCC [18]. As such, prevention and treatment of HCC migration and invasion is of great importance for improving the prognosis of HCC patients [19]. EMT plays an important role in the process of metastasis; HCC cells can obtain mesenchymal characteristics though EMT, which enhance their capacity for movement [20]. There have been many reports regarding the molecular mechanisms of EMT, and the relevant signalling pathways have been thoroughly examined; however, the relationship between the actin cytoskeleton and EMT is not yet clear. In this study, we demonstrated that CAPZA1 inhibits the metastasis of HCC cells and that its expression is negatively correlated with EMT. This confirms the hypothesis that CAPZA1 regulates EMT in HCC cells via regulation of the actin cytoskeleton, thereby inhibiting the migration and invasion of HCC cells.

CAPZ is an actin-binding protein composed of α and β subunits that was originally isolated by Isenberg et al. from Acanthamoeba, insect and bovine brain tissue [21, 22]. Previous studies on CAPZ have primarily focused on the relationship between actin filaments and cardiac hypertrophy, as well as the possibility that low CAPZ expression is related to cardiac hypertrophy [23–25]. However, there have been fewer studies on the role of CAPZ in tumours. Sun et al. reported that changes in CAPZ expression could be used as a diagnostic marker for malignant melanoma [26], and Yu et al. observed that CAPZA1 expression was reduced in stage IV neuroblastoma [27]. Lee et al. reported that CAPZA1 overexpression was a marker of good prognosis for patients with gastric cancer [14]. However, it should be noted that there have been few studies on the role of CAPZA1 in HCC.

In this study, we reported that CAPZA1 inhibits the metastasis of HCC cells. Testing 129 clinical samples, we found that CAPZA1 expression is negatively correlated with the metastasis of primary HCC, and these results were further verified in our *in vitro* and *in vivo* experiments. Subsequently, we demonstrated that CAPZA1 controlled EMT in HCC cells by regulating the actin cytoskeleton. EMT requires extensive cytoskeletal remodelling, wherein the dynamic assembly of actin filaments plays a critical role in cell movement [28, 29]. CAPZA1 can bind to the barbed ends of actin filaments where it regulates the dynamic assembly of actin filaments [30]. We found that blocking CAPZA1 expression down-regulated expression of the epithelial marker E-cadherin and up-regulated expression of the mesenchymal markers N-cadherin and Vimentin; when CAPZA1 was overexpressed, the opposite results were found. Changes in the expression of the EMT transcription factors Snail1 and ZEB1 were also observed when CAPZA1 expression was changed. CAPZ plays a regulatory role in actin filaments assembly [21]; we also validated that CAPZA1 can bind to actin filaments in our study. However, how the binding of CAPZ to actin filaments is regulated is seldom reported in tumour tissue; therefore, future studies will investigate this aspect of CAPZ activity in malignant HCC.

## Conclusions

CAPZA1 inhibits EMT in HCC cells by regulating actin filament assembly, thereby reducing the invasion and migration abilities of HCC cells. Thus, CAPZA1 could be a tumour biomarker to determine the prognosis of HCC patients.

## Abbreviations

CapZ: Capping protein
CAPZA1: Capping protein α1 subunit
CCK-8: Cell counting kit-8
EMT: Epithelial-mesenchymal transition
HCC: Hepatocellular carcinoma
IB: Immuno blot
IP: Immunoprecipitation
MD: Moderately differentiated
MRI: Magnetic resonance image
PD: Poorly differentiated
qPCR: Quantitative PCR
siRNA: Small interfering RNA
WD: Well differentiated
